# Butein inhibits oral squamous cell carcinoma growth via promoting MCL-1 ubiquitination

**DOI:** 10.7150/jca.94546

**Published:** 2024-04-15

**Authors:** Ruirui Wang, Xiaoying Li, Jidong Wang

**Affiliations:** 1 Department of Oral and Maxillofacial Surgery, Changde Hospital, Xiangya School of Medicine, Central South University (The first people's hospital of Changde City), Changde, Hunan 415000, China.; 2 Department of Radiology, The Third Xiangya Hospital of Central South University, Changsha, Hunan 410013, China.

**Keywords:** Oral squamous cell carcinoma, MCL-1, Ubiquitination, FBW7.

## Abstract

Oral squamous cell carcinoma (OSCC) is the most common malignant head and neck carcinoma type. Myeloid cell leukemia-1 (MCL-1), an anti-apoptotic BCL-1 protein, has been verified to be among the most highly upregulated pathologic proteins in human cancers linked to tumor relapse, poor prognosis and therapeutic resistance. Herein, therapeutic targeting MCL-1 is an attractive focus for cancer treatment. The present study found that butein, a potential phytochemical compound, exerted profound antitumor effects on OSCC cells. Butein treatment significantly inhibited cell viability, proliferation capacity and colony formation ability, and activated cell apoptotic process. Further potential mechanism investigation showed that promoting MCL-1 ubiquitination and degradation is the major reason for butein-mediated OSCC cell cytotoxicity. Our results uncovered that butein could facilitate E3 ligase FBW7 combined with MCL-1, which contributed to an increase in the ubiquitination of MCL-1 Ub-K48 and degradation. The results of both *in vitro* cell experiments and *in vivo* xenograft models imply a critical antitumor function of butein with the well-tolerated feature, and it might be an attractive and promising agent for OSCC treatment.

## Introduction

Oral squamous cell carcinoma (OSCC) is the most prevalent malignant tumor type in the oral cavity and oropharynx, which originates from the squamous cells lining the oral tissues, such as tongue, buccal mucosa, lips, gingiva and alveolar 1, 2. Smoking, areca nut products, alcohol, bacterial and viral infections, and unhealthy oral conditions are considered to be the pivotal etiologic factors associated with OSCC 3-5. As shown in the latest global cancer data, OSCC causes more than 370,000 new cancer cases and 170,000 cancer-related deaths 6, 7. Depending on the clinical situation of the disease, surgery, chemotherapy, radiotherapy, targeted therapy and immunotherapy are the main modalities for OSCC treatment. However, the five-year survival rate is still less than 50% due to the irresistible tumor recurrence and metastasis 8-11. Exploring the early diagnosis and effective treatment strategies remains a clinical challenge for OSCC, characterized by high mortality and poor prognosis.

Extensive research over the past few decades on natural products provides ample evidence for the safety and effectiveness of phytochemicals against a variety of different diseases including cancer 12-14. Butein is a potential phytochemical obtained from numerous plants (e.g. *Semecarpus anacardium, Dalbergia odorifera, Caragana jubata, Rhus verniciflua*), drawing widespread attention for its medicinal values against diabetes, neuropathy, inflammatory, cancer treatment and other diseases 15. Increasing lines of evidence unveiled that Butein has a high potential antitumor agent for the treatment and prevention of various cancers, such as hepatocellular carcinoma 16, breast cancer 17, colorectal cancer 18, lung cancer 19, etc., by interfering with the invasion, metastasis, proliferation, survival, angiogenesis and therapeutic resistance malignant phenotype of tumor cells 20, 21. In addition, butein has been reported in previous studies to exhibit potent antiproliferative, cytotoxic, antimigratory and invasive effects in OSCC cells 22. However, the underlying antitumor molecular mechanism of butein in OSCC is not fully understood and further studies are still needed. The fact that it might be an attractive and promising agent for the treatment of OSCC, which requires a better understanding and exploration of its pharmacological mechanisms.

In this present study, we preliminarily investigated the therapeutic potential of butein in OSCC. The effects of butein on proliferation and apoptosis in CAL27 and SCC9 cell lines were evaluated, and its antitumor activity was further demonstrated in xenograft models.

## Materials and Methods

### Cell culture and reagents

Human OSCC cell lines CAL27 and SCC9 were purchased from the American Type Culture Collection (ATCC, Manassas, VA). All cell lines were cultured in DMEM/F12 medium supplemented with 10% Fetal Bovine Serum (FBS) and maintained in a 37 °C humidified incubator at with a 5% CO_2_ atmosphere. The natural product butein and inhibitors, including MG132, cycloheximide (CHX), z-VAD-FMK, Necrostatin-1 (Nec-1), and Chloroquine (CQ) were obtained from Selleck Chemicals (Houston, TX), The inhibitor MG132, and cycloheximide (CHX) were obtained from Thermo Fisher Scientific (Waltham, MA). Antibodies against MCL-1 (#39224), ubiquitin (#3936), HA-tag (#3724), cleaved-caspase 3 (#9664), cytochrome C (#11940), Bax (#5023), VDAC1 (#4661), β-actin (#4970), and α-Tubulin (#3873) were purchased from Cell Signaling Technology, Inc. (Beverly, MA). The FBW7 (#ab109617) and ki67 (#ab16667) antibodies were obtained from Abcam (Cambridge, United Kingdom). The transfection reagent Lipofectamine^TM^ 2000 was purchased from Invitrogen (#11668019, Carlsbad CA, USA).

### MTS assay

To analysis the cytotoxic effect of butein on OSCC cells, MTS assay was performed to measure the cell viability according to the manufacturer's instructions. CAL27 and SCC9 cells were seeded in 96-well plates (3 × 10^3^ cells/well) and incubated overnight. Cells subjected to various concentrations of butein for different times. Finally, the cell viability was determined via analyzing the optical density at 450 nm of each well after adding MTS reagent (#G3580, Madison, WI).

### Colony formation assays

To evaluate the colony formation ability of CAL27 and SCC9 cells, anchorage-independent cell growth assay and plate colony formation assay were performed as described previously 23. Briefly, for anchorage-independent cell growth assay, OSCC cells were resuspended at a concentration of 8×10^3^ cells/ml and inoculated in 6-well plates containing Basal Medium Eagle (0.6% agar, 10% FBS, and different concentrations of butein. After cultured in CO_2_ incubator at 37 ℃ for 14 days, the colonies were observed and counted. For plate colony formation assay, the OSCC cells were seeded in a 6-well plate with a density of 500 cells per well. After incubating for 14 days, the samples were incubated with 4% paraformaldehyde for 30 minutes to fix, stained with crystal violet solution for 20 minutes, washed with PBS several times, dried and photographed.

### Western blotting (WB) assay

OSCC cells were treated with butein and subjected to RIPA lysis buffer to acquire whole-cell extract (WCE). The BCA protein assay kit (#23228, Thermo Fisher Scientific) was used to measure the protein concentrations, and then equal amounts of protein samples were separated on 10% SDS‒PAGE gel and transferred onto the PVDF membrane. After blocking with 5% non-fat milk for 60 min, the membranes were incubated with primary antibodies overnight at 4 ℃, then with secondary antibodies for 60 min at room temperature. The target protein band was detected with Enhanced Chemiluminescence reagent (ECL) (#34579, Thermo Fisher Scientific).

### Xenograft tumor model

The *in vivo* mice experiments followed guidelines established by the Medical Research Animal Ethics Committee, Central South University (Changsha, China). For xenograft tumor model establishment, CAL27 (2 × 10^6^) cells were subcutaneously injected into the right flank of 6-week-old athymic nude mice (n = 6). Tumor volume (length × width × width/2) and mouse weight were measured every two days. When tumor volume reached around 100 mm^3^, intraperitoneal administration of butein (10 mg/kg/ in 100 µL Corn oil every 2 days) or vehicle control (0.5% dimethyl sulfoxide in 100 µL Corn oil /every 2 days) was done. The tumor-bearing mice were euthanized with CO_2_ (3 L/min) for 5 min after the tumor volume reached about 800 mm^3^, followed by tumor tissue collection for recording weight and immunohistochemical staining, serum collection for analyzing the level of white blood corpuscles (WBC), red blood corpuscles (RBC), Hemoglobin (Hb), blood urea nitrogen (BUN), aspartate aminotransferase (AST), and alanine aminotransferase (ALT). Subsequently, the tissues of the spleen, kidney, lung, heart, and liver were obtained and fixed using 4 % formaldehyde for Hematoxylin and eosin (H&E) staining.

### Statistical analysis

GraphPad Prism Software was used to analyze statistical significance. Data were represented as mean ± standard deviation (SD) with at least three independent determinations. The student's t-test and one-way ANOVA were used to analyze statistics between different groups. A probability value of p < 0.05 was set as the significance threshold for a statistically significant difference.

## Results

### Butein exerts antitumor effect on OSCC cells *in vitro*

**Figure 1:**
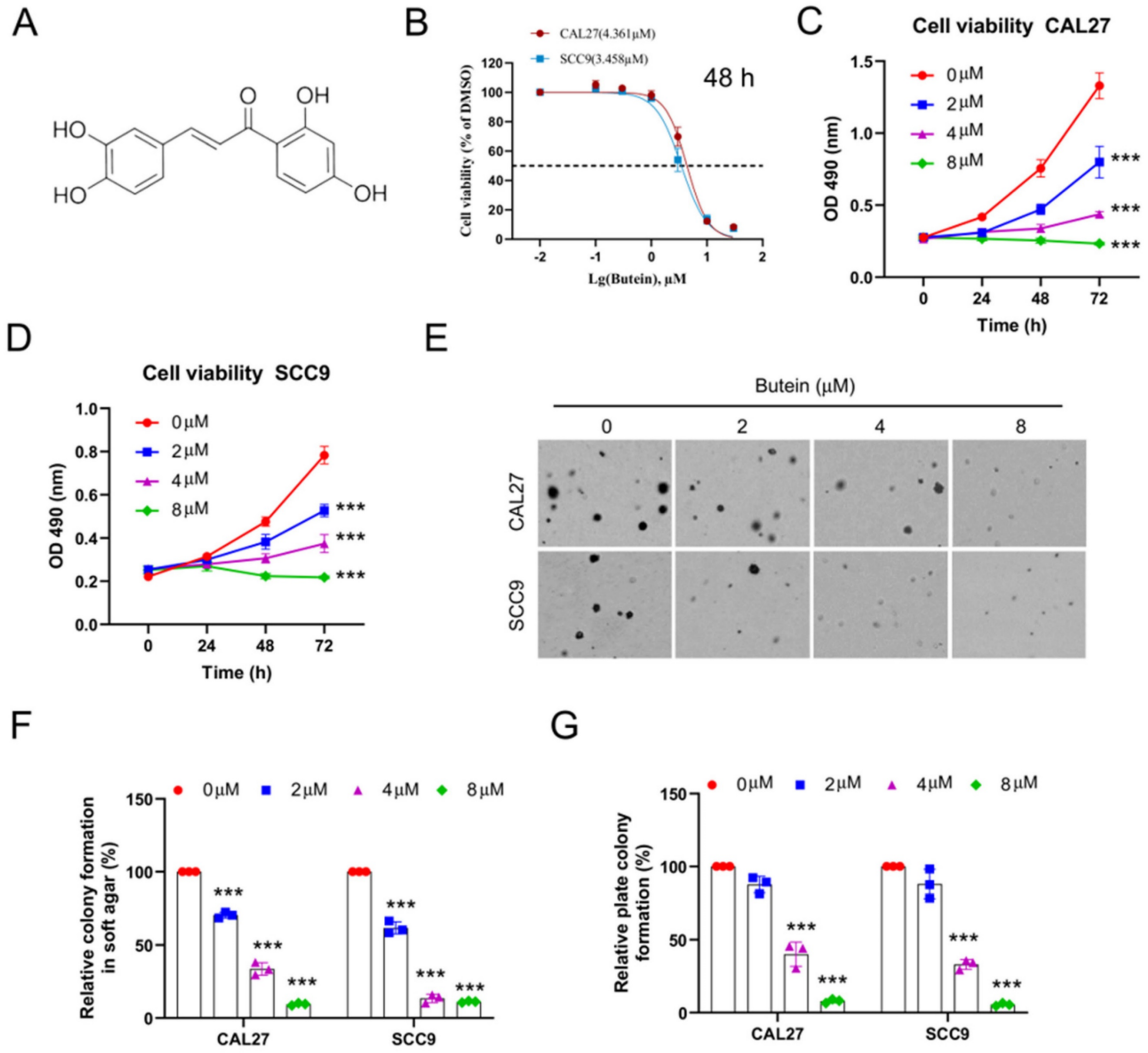
** Butein exerts antitumor effect on OSCC cells *in vitro*.** A. Chemical structure of butein. B. CAL27 and SCC9 cells were treated with different concentrations of butein for 48 h. MTS assay was performed to detect the IC_50_ value of butein. C and D. CAL27 (C) and SCC9 (D) cells were treated with butein (0/2/4/8 µM) for various times, and cell viability was determined by MTS assay. E-G. CAL27 and SCC9 cells were treated with butein (0/2/4/8 µM) for 48 h. Soft agar assay was used to detect the anchorage-independent growth capacity (E and F); Plate colony formation assay was used to analyze colony formation viability (G).

To investigate the pharmacological activity of butein (Figure 1A) on OSCC cells *in vitro*, CAL27 and SCC9 cell lines were selected. Firstly, half maximal inhibitory concentration of butein was determined, and cell viability of CAL27 and SCC9 cells was inhibited with 50% inhibitory concentration (IC50) values of 4.361 μM and 3.458 μM, respectively (Figure 1B). Then, OSCC cells were subjected to butein treatment with different concentrations for 0 h, 24 h, 48 h and 72 h. The data analysis suggested that cell viability of CAL27 and SCC9 cells was inhibited in a dose-dependent manner (Figure 1C and D). Moreover, it was found that the colony formation capacity of CAL27 and SCC9 cells was impaired after being treated with butein using soft agar assay and plate colony formation assay (Figure 1E-G). Our studies discovered butein may possess a novel antitumor pharmacological activity on OSCC cells.

### Butein induces MCL-1 degradation by promoting its ubiquitination

**Figure 2:**
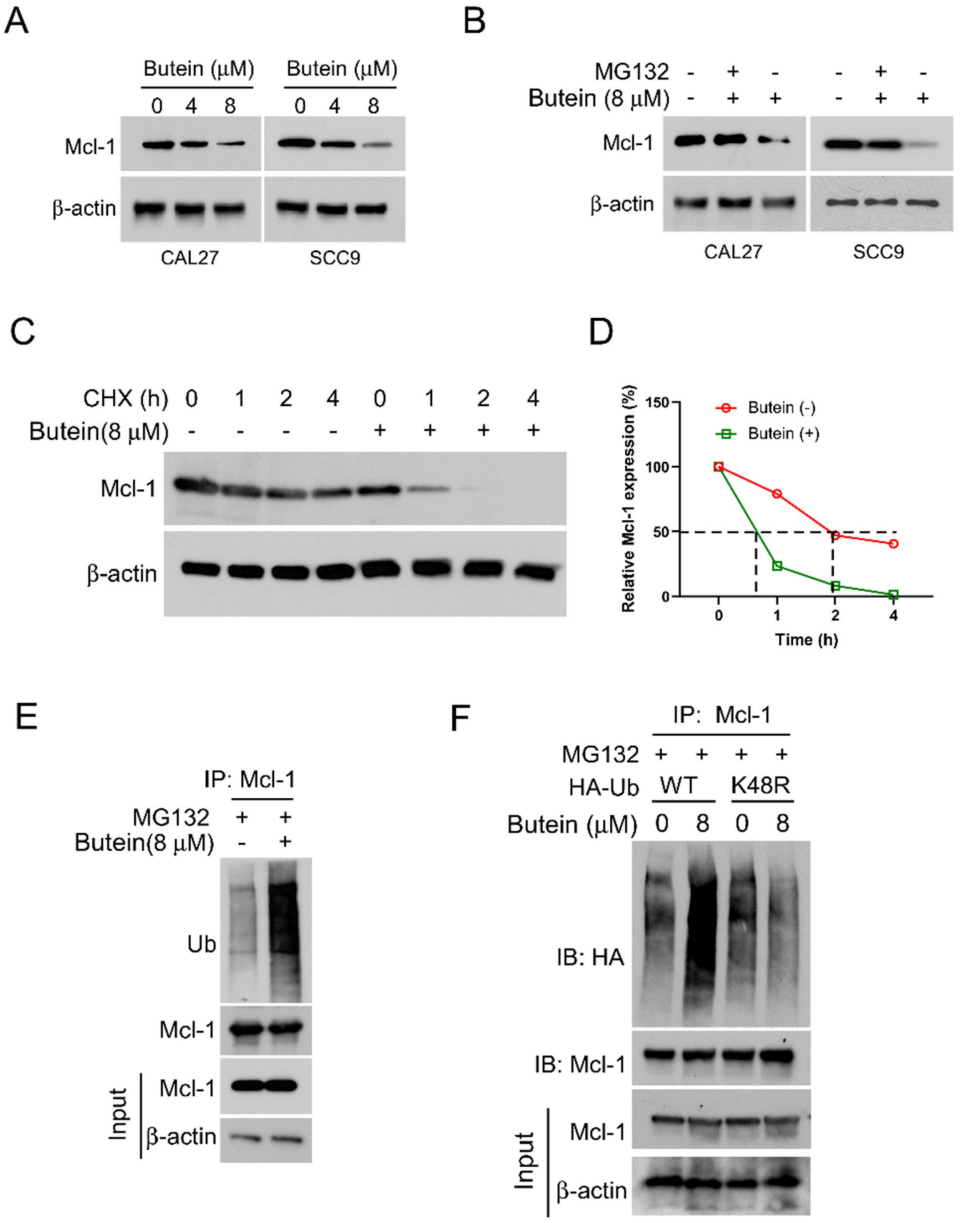
** Butein induces MCL-1 degradation via promoting its ubiquitination.** A. CAL27 and SCC9 cells were treated with butein (0/4/8 µM) for 48 h. WB was used to detect the expression of MCL-1 protein. B. CAL27 and SCC9 cells were treated with butein (8 µM) for 48 h, and incubated with MG132 (20 µM) for 8 h, WB was used to detect the expression of MCL-1 protein. C and D. CAL27 cells were treated with/without butein (8 µM) for 48 h, and followed by CHX treatment for various times. WB was used to examine the expression of MCL-1 protein. E. CAL27 cells were treated with butein (8 µM) for 48 h, and incubated with MG132 (20 µM) for 8 h, IP and WB assay was used to determine the ubiquitination level of MCL-1 protein. F. CAL27 cells were transfected with WT-Ub or K48R-Ub for 24 h, and then treated with butein (8 µM) for 48 h, and MG132 (20 µM) for 8 h, IP and WB assay was used to analyze the ubiquitination level of MCL-1 protein.

In order to explore the potential molecular mechanisms of the anti-tumor effect of butein, we initially examined the expression levels of the critical cancer-related protein MCL-1. Following treatment with different concentrations of butein, our results showed a concentration-dependent downregulation of MCL-1 protein level in CAL27 and SCC9 cells (Figure 2A). Importantly, when protein proteasome inhibitor MG132 was administered simultaneously, the butein-induced downregulation of MCL-1 was alleviated (Figure 2B). Subsequently, we investigated whether butein has an impact on the half-life of MCL-1 protein. We treated CAL27 cells with a protein synthesis inhibitor, cycloheximide (CHX), at different time points, and the Western blot results indicated a significant acceleration in the degradation rate of MCL-1 protein after butein treatment (Figure 2C and D). The preliminary research results suggest that the butein-mediated downregulation of MCL-1 is associated with ubiquitin-proteasome degradation. As shown in Figure 2E, ubiquitination analysis confirmed that the ubiquitin level of protein MCL-1 increased significantly after butein treatment. Furthermore, HA-Ub WT or K48R mutant plasmids were transfected into CAL27 cells, and the results showed a significant reduction in the ubiquitination level of MCL-1 in the K48R mutant group compared to the WT group (Figure 2F). These results indicated that butein-induced MCL-1 downregulation is related to the regulation of ubiquitination.

### E3 ligase FBW7 is necessary for butein-induced MCL-1 ubiquitination

**Figure 3:**
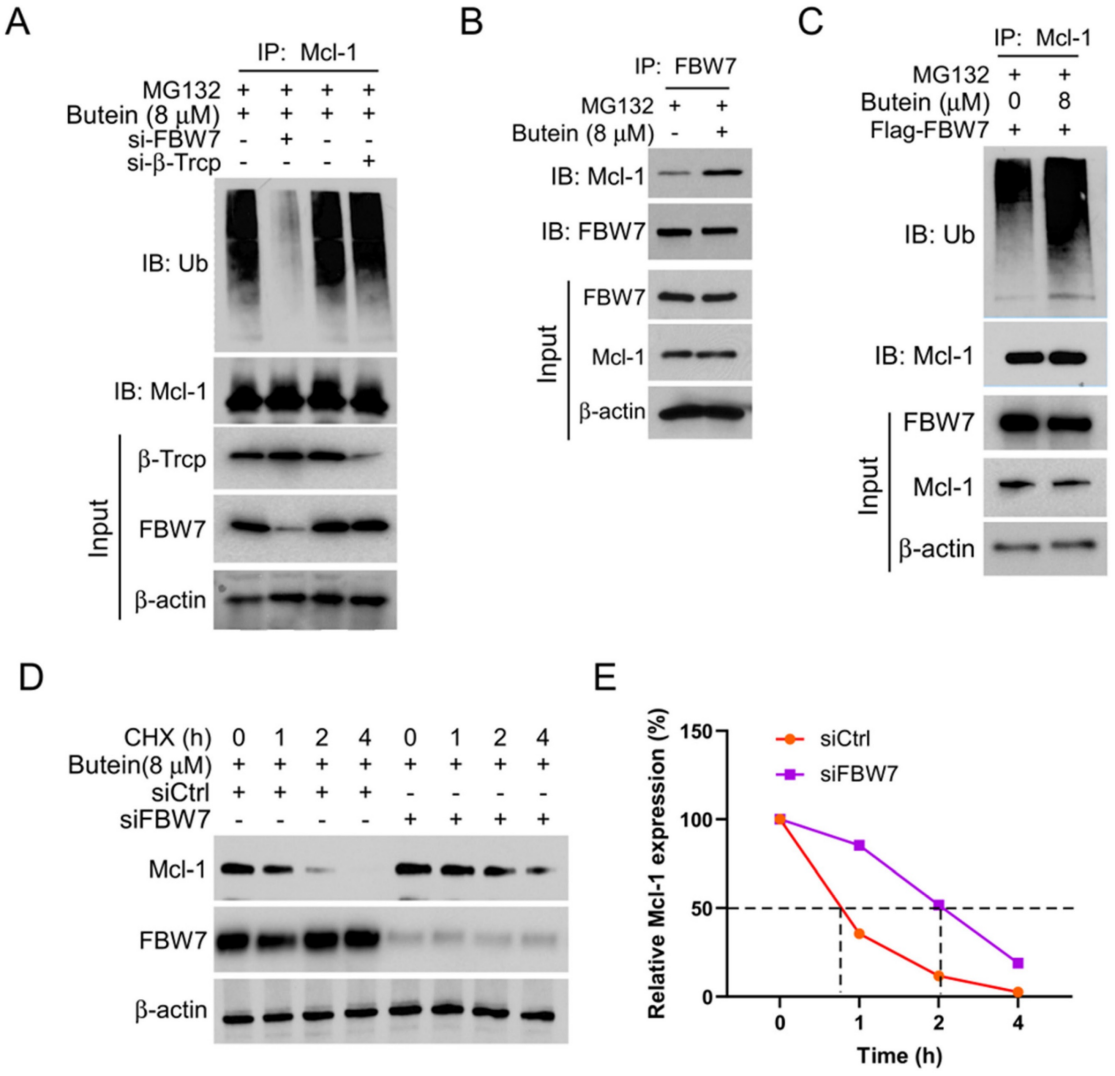
** E3 ligase FBW7 is necessary for butein-induced MCL-1 ubiquitination.** A. CAL27 cells were transfected with si-FBW7 or si-β-Trcp shRNA for 24 h, and then treated with butein (8 µM) for 48 h, and MG132 (20 µM) for 8 h, IP and WB assay was used to analysis the ubiquitination level of MCL-1 protein. B. CAL27 cells were treated with/without butein (8 µM) for 48 h, and MG132 (20 µM) for 8 h. Co-IP and WB assay was used to detect the interaction between MCL-1 and FBW7. C. CAL27 cells were transfected with Flag-FBW7 plasmid for 24 h, and then treated with/without butein (8 µM) for 48 h, and MG132 (20 µM) for 8 h, IP and WB assay was used to analyze the ubiquitination level of MCL-1 protein. D and E. CAL27 cells were transfected with si-FBW7 or si-Ctrl shRNA for 24 h, and then treated with butein (8 µM) for 48 h, followed by CHX treatment for various times. WB was used to examine the expression of MCL-1 protein.

Previous studies have found that some E3 ligases, such as FBW7 and β-Trcp, are involved in post-translational ubiquitin modification of MCL-1 24, 25. In this study, we explored whether FBW7 and β-Trcp are associated with butein-induced MCL-1 degradation. The results showed that transfection with siRNA to knockdown FBW7, but not β-Trcp, significantly reduced the ubiquitination level of MCL-1 (Figure 3A). Immunoprecipitation experiments further verified that the interaction between MCL-1 and E3 ligases FBW7 increased remarkedly following treatment with butein (Figure 3B). Consistently, we found that the ubiquitination level of MCL-1 was upregulated with butein treated under exogenous transfection with Flag-FBW7 plasmid for overexpression (Figure 3C). Next, protein half-life experiments further confirmed that FBW7 is involved in the ubiquitin-proteasome degradation of MCL-1. As shown in Figures 3D and E, knocking down FBW7 can delay the rate of butein-induced MCL-1 degradation. In summary, our study results suggested that FBW7 plays an essential role in butein-mediated MCL-1 ubiquitin-proteasome degradation.

### Butein-induced MCL-1 degradation activates intrinsic apoptosis

**Figure 4:**
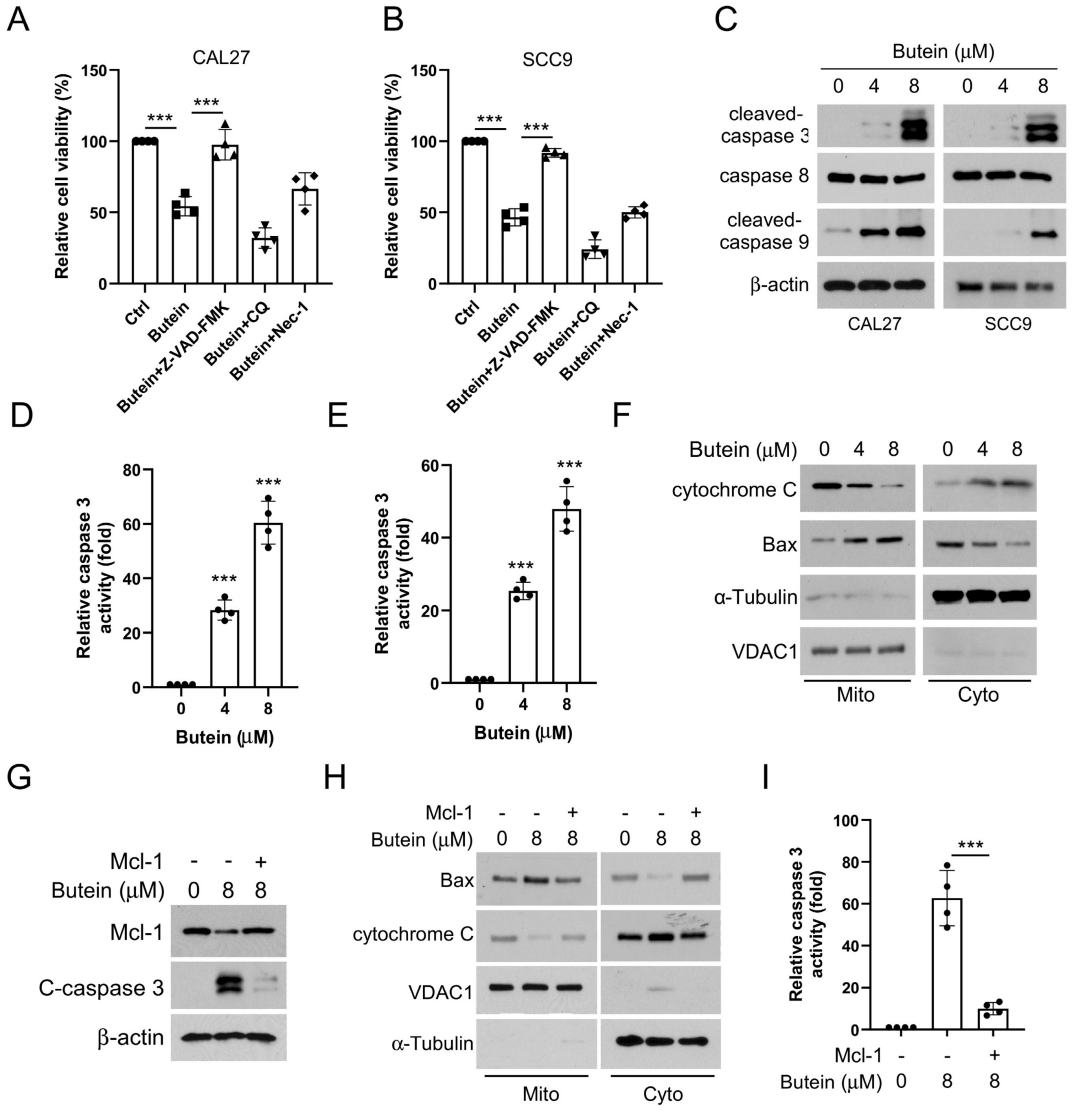
** Butein-induced MCL-1 degradation activates intrinsic apoptosis.** A and B. CAL27 and SCC9 cells were pre-treated with z-VAD-FMK, CQ, or Nec-1 for 4 h, and then treated with butein for 48 h. C-E. CAL27 and SCC9 cells were treated with butein (0/4/8 µM) for 48 h. WB was used to detect the expression of cleaved-caspase 3, caspase 8, cleaved-caspase 9 protein (C); Caspase 3 Assay Kit was used to examine caspase 3 activity (D and E). F. CAL27 cells were treated with butein (0/4/8 µM) for 48 h, subcellular fractions were isolated to detect the expression of cytochrome c and Bax using WB assay. G-I. CAL27 cells were transfected with MCL-1 plasmid and treated with butein (8 µM) for 48 h. WB assay was used to detect the expression of cleaved-caspase 3 protein (G); Subcellular fractions were isolated to detect the expression of cytochrome c and Bax using WB assay (H); Caspase 3 Assay Kit was used to examine caspase 3 activity (I).

In view of the fact that MCL-1 serves as a vital member of the anti-apoptotic family, consistent with our study, we found that the cell viability of OSCC cells was significantly restored in the experimental group treated with apoptosis inhibitor z-VAD-FMK, rather than autophagy inhibitor chloroquine and necrosis inhibitor necrostatin-1 (Figure 4A and B).

WB experiments showed that the protein expression level of the cleaved-caspase 3 and cleaved-caspase 9 increased in a concentration-dependent manner in CAL27 and SCC9 cells treated with butein (Figure 4C). However, the protein level of caspase 8 was unchanged, indicating that butein activates intrinsic apoptosis (Figure 4C). We next assayed caspase 3 activity using a caspase 3 assay kit, which showed that caspase 3 activity was significantly upregulated in butein-treated CAL27 and SCC9 cells (4D and E). Analysis of cell subcomponents cytoplasm and mitochondria showed that the release of cytochrome c from mitochondria to the cytoplasm and the accumulation of Bax from the cytoplasm to mitochondria was enhanced with butein treatment, which led to triggering apoptosis (Figure 4F). Noteworthily, overexpression of MCL-1 neutralized the butein-induced upregulation of c-caspase 3 expression and activity (Figure 4G and I) and prevented the release of cytochrome c into the cytoplasm and the accumulation of Bax in mitochondria (Figure 4H). Together, these results implied that the antitumor activity of butein is associated with the activation of intrinsic apoptosis through the downregulation of MCL-1 expression.

### Butein inhibits tumor growth of OSCC cells *in vivo*

**Figure 5:**
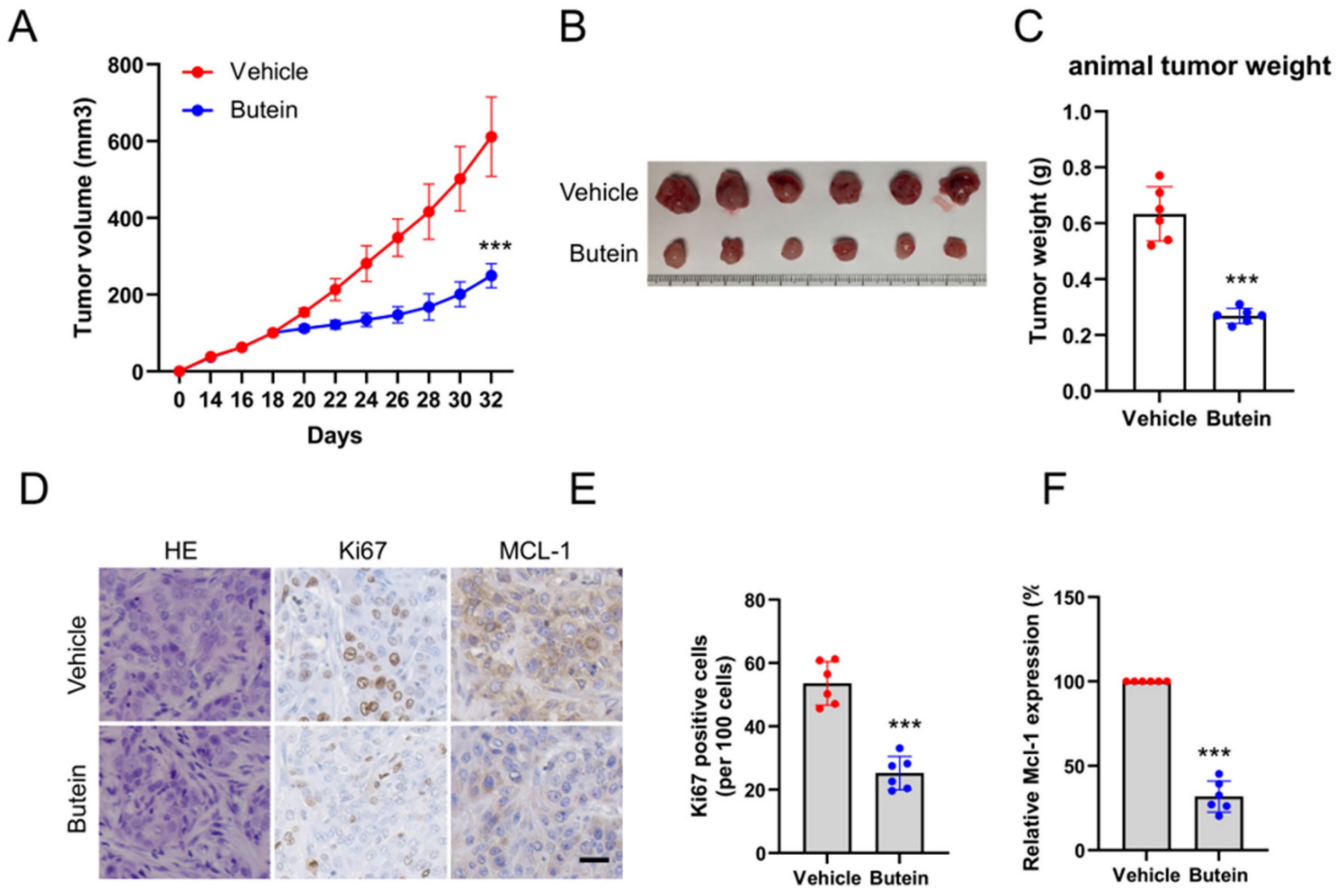
** Butein inhibits tumor growth of OSCC cells *in vivo*.** A-C. The tumor volume (A), the image of tumor mass (B), and tumor weight (C) of CAL27-derived xenograft tumors (n = 6) with vehicle or butein treatment. D. IHC staining of MCL-1 and Ki67 in CAL27-derived xenograft tumor sections treated with vehicle or butein. Scale bar, 25 μm. E and F. Qualification analysis of Ki67 (E) and MCL-1 (F) in CAL27-derived xenograft tumor sections treated with vehicle or butein.

**Figure 6:**
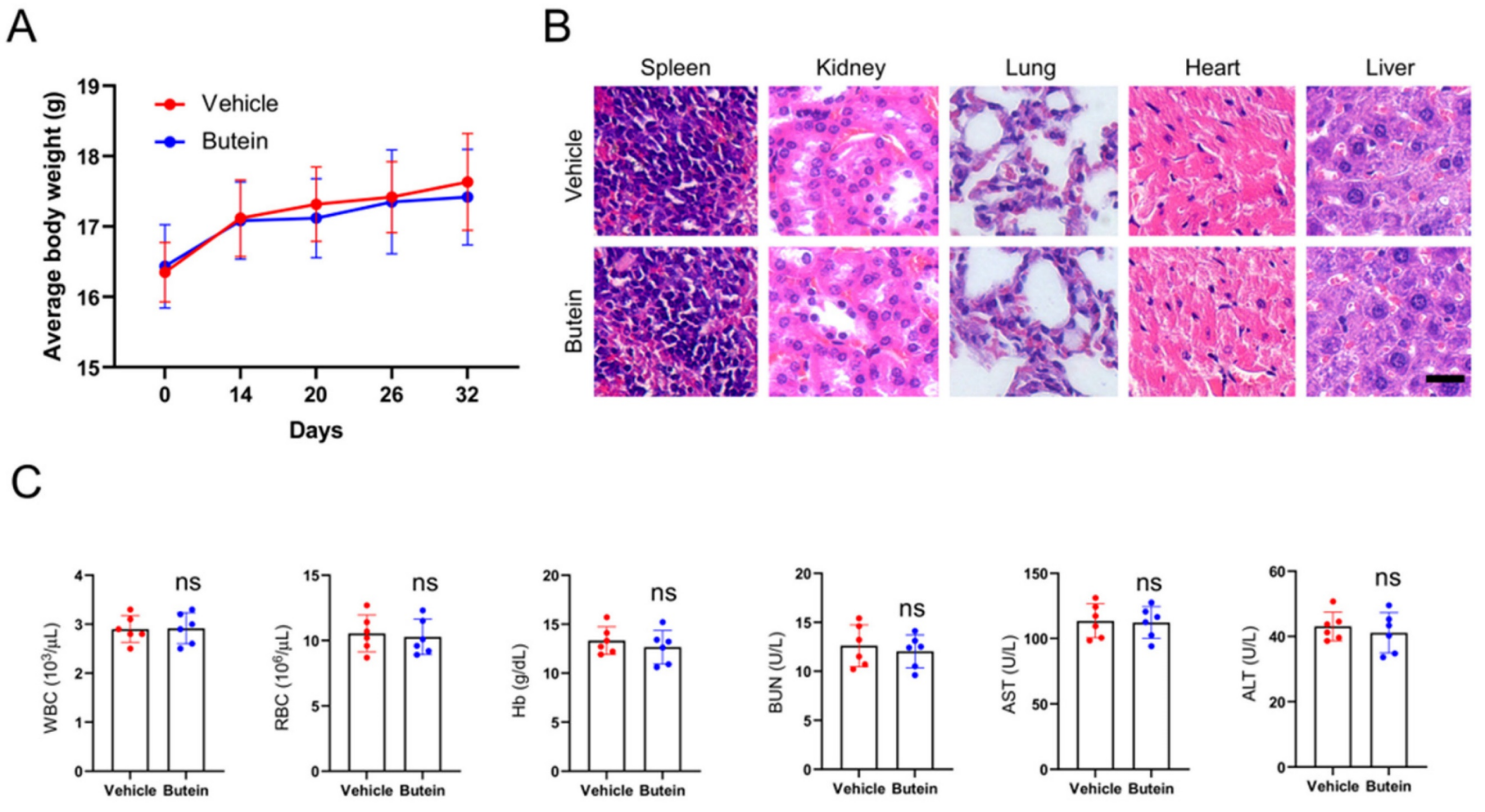
** Toxicity analysis for butein treatment *in vivo*.** A. Body weight recording of CAL27-derived xenograft tumor-bearing mice treated with vehicle or butein. B. HE staining of kidney, lung, heart, and liver sections of CAL27-derived xenograft tumor-bearing mice treated with vehicle or butein. C. Blood analysis of WBC, RBC, Hb, BUN, AST, and ALT level of CAL27-derived xenograft tumor-bearing mice treated with vehicle or butein.

The xenograft mouse models were established with CAL27 cells to explore the therapeutic effects of butein on subcutaneous tumors. When subcutaneous tumor volume reached around 100 mm^3^, butein was injected intraperitoneally in a dose of 10 mg/kg every 2 days, and the vehicle group was administrated with 0.5% DMSO. It was found that the tumor volume and weight in the butein-treated group were significantly smaller than that in the vehicle group (Figure 5A-C). In addition, the tumor tissue sections were subjected to IHC analysis to detect the expression of cell proliferation molecule Ki67 and MCL-1. The experimental results showed that the Ki67 and MCL-1 protein staining in the butein-treated group was weaker than in the vehicle group (Figure 5D-F). Furthermore, we evaluated the tolerance of butein *in vivo* and found no significant difference in body weight between the butein-treated and the control groups (Figure 6A). Simultaneously, the major organs, including the spleen, kidney, lung, heart, and liver, and blood samples were collected for pathological analysis. Compared with the vehicle group, the H&E staining of major organ tissues displayed no observable pathological changes upon treatment with butein (Figure 6B). The blood and serological analysis results showed that the concentration of WBC, RBC, Hb, BUN, AST and ALT between the butein-treated and vehicle groups was no significant difference (Figure 6C). Overall, *in vivo* experimental results suggested that butein exerted an effective antitumor activity in OSCC cells with a well-tolerated.

## Discussion

Oral squamous cell carcinoma, the most common malignant head and neck carcinomas, is one of the leading causes of tumor-related mortality 26. Accumulating researches have been devoted to shed light on the molecular mechanisms related to the occurrence and progression of OSCC 27-30. Previous studies found that TAF1L, a TAF1 homologue, was upregulated in OSCC cell lines and activated autophagy to regulate OSCC cells to escape apoptosis for promoting OSCC progression 31. Dong et al. identified a novel RNA-splicing-related gene, Lsm12 might be a pivotal biomarker in OSCC via regulating the alternative splicing of USO1 exon 15, which was closely related to the malignant phenotypes of OSCC cells 32. A recent study discovered that the expression of DUXAP9, a novel nuclear-localized lncRNA, was highly associated with cell proliferation, invasion and migration of OSCC cells by suppressing CDK1-mediated EZH2 degradation, suggesting that DUXAP9 might be a promising target for OSCC treatment 33. MCL-1 is one of the antiapoptotic members of the BCL-2 family, and it has been verified that MCL-1 expression level was significantly upregulated in OSCC tumor tissues and is linked to poor outcome, therapeutic resistance, and disease progression of oral cancers 34-37.

Therefore, discovering antitumor agents targeting these oncoproteins is an attractive strategy for OSCC treatment. In this study, it turned out that the butein was sensitive to OSCC cell lines CAL27 (IC_50_=4.361 μM) and SCC9 (IC_50_=3.458 μM). Interestingly, the MTS and clone formation assay demonstrated that cell viability and proliferation of CAL27 and SCC9 cells were significantly inhibited upon butein treatment in a dose-dependent manner. Mechanistic study demonstrated that the protein expression of MCL-1 was downregulated *in vitro* and *in vivo* experiments following butein treatment, indicating that MCL-1 may serve as a candidate target protein for butein-mediated OSCC cells cytotoxicity.

*Mcl-1* gene, a homologous of the BCL-2 gene, encodes the full-length MCL-1 protein and is one of the most amplified genes in malignant tumors 38-40. MCL-1 protein is responsible for the survival of diverse cell types, and exhibits an antiapoptotic function by binding and sequestering multi-domain BH effector proteins to inhibit mitochondria cytochrome c release and mitochondrial outer membrane permeabilization (MOMP) 41-43. MCL-1 protein was upregulated among many tumors (e.g. breast cancer, colon cancer, multiple myeloma). Existing evidences shows that MCL-1 is closely related to tumorigenesis, immortalization, recurrence, and therapeutic resistance 44-46. Martina et al. found that the Mcl-1 gene is necessary for senescent tumor cell proliferation and metastatic dissemination using single-cell RNA-sequencing analysis 47. A previous study revealed that MCL-1 plays a pivotal role in regulating fatty acid oxidation (FAO) pathway proteins independently of its anti-apoptotic function in MCL-1-driven hematologic cancer cells 48. Targeting MCL-1 in breast ecosystems has been reported to induce cancer cell death and reverse the tumorigenic activation of fibroblasts 49. The important role of MCL-1 in facilitating tumor progression makes it an attractive biomarker for cancer treatment.

A great deal of research has been devoted to exploring the regulatory mechanism of MCL-1 in the processes of transcription, translation, and post-translation modification 50. To date, an increasing number of inhibitors targeting MCL-1 according to its modulational mechanism have been discovered in preclinical studies, some of which have advanced into clinical trials 51, 52. Previous studies had shown that the CDK 7/9 inhibitors SNS-032, roscovitine and flavopiridol downregulated indirectly MCL-1 protein through regulating *Mcl-1* gene transcription 53, 54. A several of compounds, such as S64315, AZD5991, AMG and ABBV-467, have entered phase 1 clinical trials with highly selective inhibition MCL-1 expression 55-57. In the present study, we discovered a natural product, butein, markedly accelerated MCL-1 degradation and downregulated the expression of MCL-1 protein in OSCC cell lines. Moreover, the expression and activity of cleaved-caspase 3, a classical biomarker of intrinsic apoptosis activation, were significantly increased after butein treatment. While exploring the potential mechanism, the results revealed that butein-induced MCL-1 degradation was related to ubiquitin modification. At present, several E3 ubiquitin ligases, such as Trim17, APC/CCdc20, FBW7 and β-TrCP, and deubiquitinase USP9x have been demonstrated to regulate MCL-1 post-translational ubiquitin modification 41. In this study, our findings discovered that butein-mediated upregulation of MCL-1 ubiquitination is due to enhanced the interaction between MCL-1 and FBW7.

In summary, this study underscored an underlying mechanism of natural compound butein-induced inhibition of OSCC cells. Butein exhibited an antitumor property of significantly suppressing cell proliferation and activating intrinsic apoptosis via enhancing the binding of FBW7 and MCL-1 to promote MCL-1 ubiquitination and degradation. Our discoveries imply that butein holds the potential as a feasible candidate for targeting MCL-1 in OSCC treatment.

## Funding

This work was supported by the Central South University Graduate Research Innovation Project (No. 1053320220808).
